# Nephrotic syndrome associated with Kimura’s disease: a case report and literature review

**DOI:** 10.1186/s12882-018-1123-y

**Published:** 2018-11-08

**Authors:** Song Ren, Xin Yi Li, Fang Wang, Ping Zhang, Yuan Zhang, Gui Sen Li, Li Wang, Xiang Zhong

**Affiliations:** Department of Nephrology, Sichuan Academy of Medical Sciences & Sichuan Provincial People’s Hospital, School of Medicine, University of Electronic Science and Technology of China, No.32, West Section 2, Ring 1 Road, Chengdu, 610072 Sichuan China

**Keywords:** Kimura’s disease, Minimal change disease, Nephrotic syndrome, Prednisone

## Abstract

**Background:**

Kimura’s disease (KD) is a rare chronic inflammatory disorder with a high incidence of renal involvement. In this report, we present a case study of KD-associated nephrotic syndrome combined with minimal change disease (MCD) and acute renal tubular injury. Meanwhile, the clinical and histopathological characteristics of 26 patients with KD presenting with renal involvement were retrospectively evaluated.

**Case presentation:**

Here, we report a case study of a 59-year-old male patient with KD confirmed by a lymph node biopsy. He developed widespread edema and decreased urine output. A palpable swollen mobile and non-tender lymph node behind the left ear was observed upon admission. A renal biopsy revealed minimal-change lesions and acute renal tubular injury. The patient received hemodialysis because of the oliguria and renal insufficiency, and an initial dose of 40 mg/d methylprednisolone and then continued treatment with 40 mg/d prednisolone. He exhibited a good clinical response to the steroid after 6 weeks of treatment. Of the other 26 patients included in the review, 13 patients presented with mesangial proliferative glomerulonephritis, 4 with membranous nephropathy, 3 with MCD, 3 with focal segmental glomerulosclerosis, 2 with IgA nephropathy and 1 with acute tubular injury. With the exception of 2 patients who progressed to end-stage renal disease and received hemodialysis, the majority of patients responded well to treatment with corticosteroids alone.

**Conclusions:**

MCD combined with acute renal tubular injury is rare in patients with KD presenting with renal involvement. Corticosteroids may be a beneficial treatment for renal injury in patients with KD.

## Background

Kimura’s disease (KD), a rare chronic inflammatory disorder whose etiology is unknown, was first reported by Kim and Szeto in 1937.The disease has a higher prevalence in Asians [1]. KD typically presents as painless unilateral cervical lymphadenopathy or as subcutaneous masses in the head or neck region. The parotid glands and periauricular, axillary and inguinal lymph nodes are commonly affected [2, 3]. KD is diagnosed based on the presence of a characteristic subcutaneous mass or palpable lymph node in conjunction with peripheral blood eosinophilia and elevated blood IgE concentrations [4]. Some patients may display renal involvement. The histological changes in the kidney associated with KD are heterogeneous and may be consistent with minimal change disease (MCD), membranous nephropathy, focal segmental glomerulosclerosis and IgA nephropathy [5–8].This paper presents a case of KD with MCD combined with acute renal tubular injury.

## Case report

### Clinical features and laboratory findings

A 59-year-old male patient presented with widespread edema and decreased urine output. The patient had no history of active arthritis, hemoptysis, bleeding, purpura, fever, chills, weight loss, streptococcal infection or known tropical disease and had not suffered from asthma or any other atopic diseases. At the time of his visit to our hospital, his blood pressure was 136/93 mmHg, and his temperature was 36.9 °C. The physical examination showed that the patient was suffering from left-sided hearing loss and revealed the presence of a palpable swollen mobile and non-tender lymph node with a size of approximately 1.5*1 cm located behind the left ear. No other superficial lymph nodes were palpable. A urinalysis showed 3+ proteinuria and 3.68 g of proteinuria in 24 h.The serum albumin (Alb) concentration was 11.3 g/L, the serum creatinine concentration was 218.7 μmol/L, the BUN concentration was 25.33 mmol/L, the eGFR was 27.4 mL/min/m^2^, and the ESR was 112 mm/h. The patient’s serum complement (C3 and C4) levels, antinuclear antibody titers, antistreptolysin O titers, and hepatitis screening results were normal. Hematology revealed a normal hemoglobin concentration and platelet count, a total white blood cell count of 6.98*10E9/L, and a percentage of eosinophils of 6.6%. The serum IgE concentration was elevated, as it was higher than 4000 IU/mL. A *Mycobacterium tuberculosis* γ-interferon release test was negative, and a bone marrow biopsy did not display obvious abnormalities. A neck CT showed that the cervical vascular sheath was surrounded by several small lymph nodes, and an ultrasound demonstrated the presence of bilateral pleural effusions and ascites. Pleural effusion examination revealed a karyocyte count of 76*10E6/L, a neutrophil count of 12%, a lymphocyte percentage of 85%, an Alb concentration of 1.8 g/L, a lactate dehydrogenase (LDH) concentration of 44 U/L, and an adenosine deaminase (ADA) concentration of 1.3 U/L.

### Lymph node histological features

**Fig. 1:**
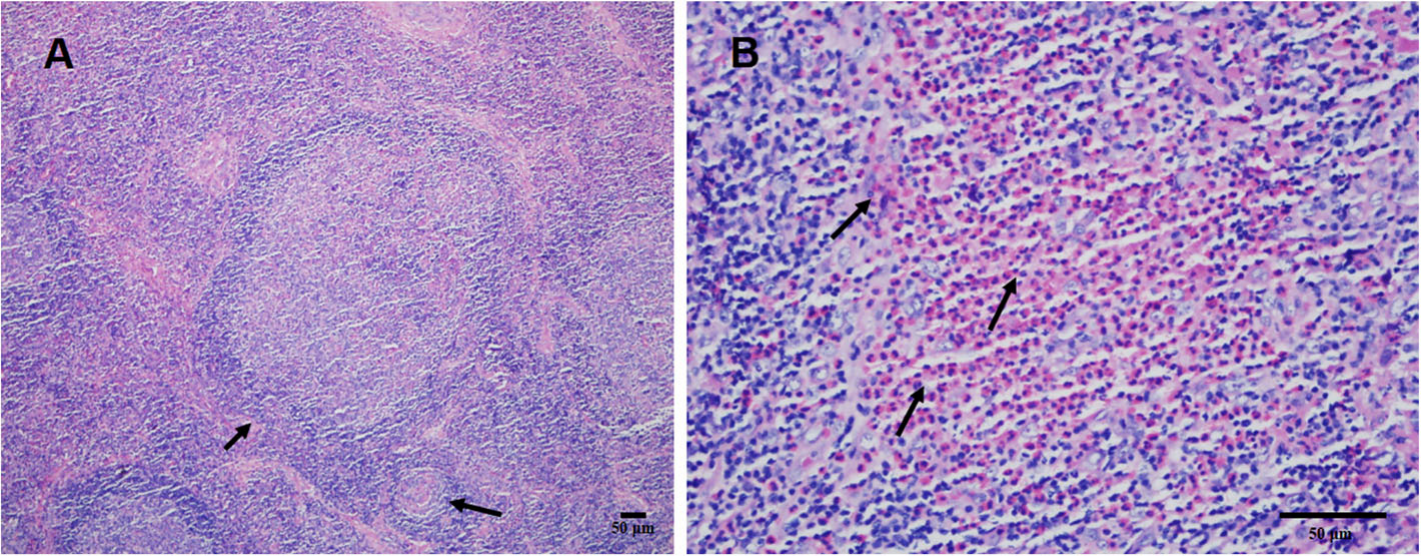
Histological examination of a lymph node biopsy. **a**. The micrograph shows reactive follicles and vascular proliferation (arrow; H&E staining × 100); **b**. Image showing eosinophil in filtration and the formation of an eosinophilic microabscess (arrow; H&E staining × 400)

Histological sections of lymph nodes were examined in the department of pathology. The examinations revealed the presence of follicular and interfollicular hyperplasia and the proliferation of venules surrounded by infiltrating eosinophils, lymphocytes, plasma cells, and mast cells. An eosinophilic microabscess was detected in the cortical area of the lymph nodes (Fig. 1). These findings confirmed the diagnosis of KD.

### Renal histology

**Fig. 2:**
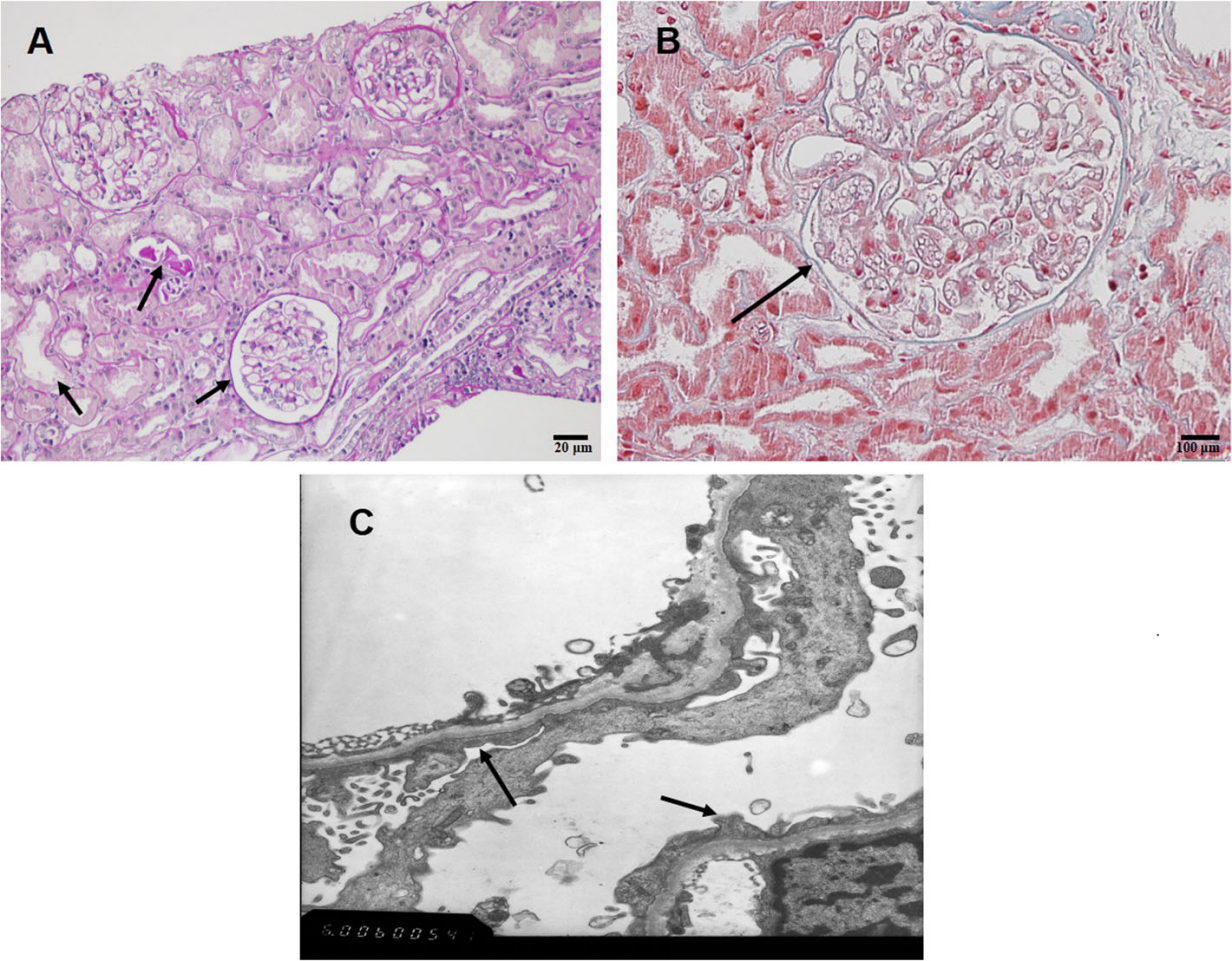
Histological examination of a renal biopsy. **a**. Light microscopy image showing the basically normal of the glomerulus with a flattened renal tubular epithelium, a slightly dilated lumen, and the presence of a few protein cysts in the dilated tubules (arrow; H&E staining × 200). **b**. Image of a minor glomerular lesion(arrow; H&E staining × 400). *C. electron* microscopy image showing mild hyperplasia of the mesangial cells and stromal cells and the extensive fusing of podocyte processes of the epithelial cells (arrow)

Renal biopsy showed that two glomeruli were sclerotic (13 glomeruli were assessed). The remaining glomerular mesangial cells and stromal cells showed signs of mild proliferation. No significant widening of the mesangial area was observed, and the basement membrane had no obvious lesions. The tubular epithelial cells displayed signs of vacuolization. Additionally, part of the renal tubular epithelium had flattened, and the bristles had fallen off. Small numbers of protein casts were present in the dilated tubules. No obvious interstitial inflammatory cell infiltration (including eosinophil infiltration) was present, and no IgG, IgA, IgM, C3, C1q, C4 or Fib deposition was noted in any of the glomeruli. Electron microscopy showed mild hyperplasia of mesangial cells and stromal cells, extensive fusing of the podocyte processes of the epithelial cells, and a lack of electron-dense deposits (Fig. 2).These findings confirmed the diagnosis of MCD combined with acute renal tubular injury.

### Treatment and follow-up

The patient’s volume load did not respond to diuretics, and his urine output decreased. Furthermore, increases in the patient’s urea and creatinine levels were observed. Therefore, hemodialysis was initiated to improve the patient’s edema and kidney function. The renal biopsy demonstrated the presence of MCD combined with acute renal tubular injury. The patient was ultimately diagnosed with KD associated with MCD and acute renal tubular injury. The patient subsequently received 40 mg/day methylprednisolone for 3 weeks. Urinary protein and renal function were monitored during treatment, which decreased the patient’s serum creatinine level to 62.3 μmol/L, increased the eGFR to 103 mL/min/m^2^, increased the serum albumin concentration to 15.6 g/L and significantly increased urine output compared with pre-treatment values. However, the patient’s proteinuria increased to7.02 g/day.

**Fig. 3:**
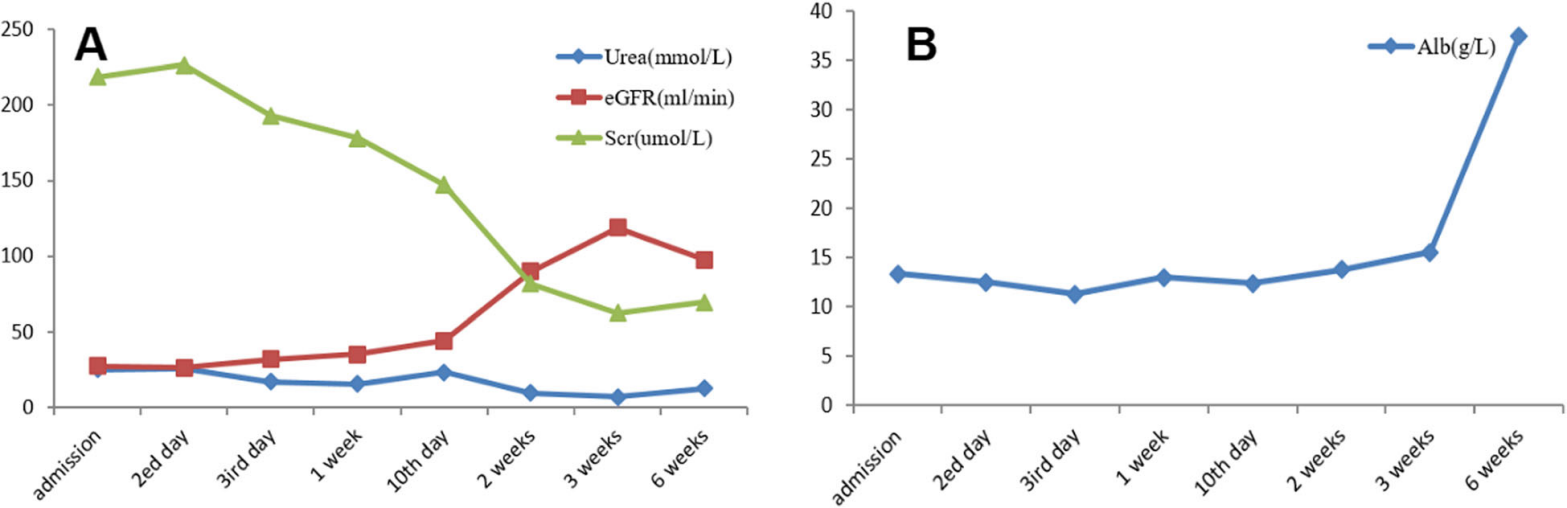
Serial biochemical changes observed in the patient. **a**. The change in renal function. After 6 weeks of treatment, the urea nitrogen concentration decreased to 12.55 mmol/L, the serum creatinine concentration decreased to 69.4 μmol/L and the eGFR increased to 97.8 mL/min. **b**. The change in serum albumin concentrations. The serum albumin concentration increased to 37.5 g/L after 6 weeks of treatment

The patient withdrew from hemodialysis and continued taking 40 mg/day prednisolone after discharge, but returned to our hospital after 6 weeks. At that time, urinalysis revealed 1+ proteinuria. The serum albumin concentration was 37.5 g/L, the serum creatinine concentration was 69.4 μmol/L, the BUN concentration was 12.55 mmol/L, and the eGFR was 97.8 mL/min/m^2^. The changes in the patient’s laboratory indexes are shown in Fig. 3**.**

### Literature review

Several databases, including PubMed, EMBASE, MEDLINE (Ovid), the Chinese National Knowledge Infrastructure (CNKI), the VIP Database for Chinese Technical Periodicals (VIP), and WanFang Data, were systematically searched from the date of database inception until Nov 01, 2017 to collect published international and domestic studies with the term “Kimura’s disease” and “Nephrotic syndrome”. The selection criteria were as follows: 1). studies including patients who were diagnosed with KD by lymph node or mass biopsy; 2). studies in which renal involvement was noted in all patients, and renal biopsy was used to define the pathology. The exclusion criteria were as follows: 1). studies which the patients were diagnosed with Kimura’s disease but with no renal involvement; 2). the data provided by the articles could not be accessed, or the full text could not be transformed. 3). the articles were reviews or abstracts. Ultimately, 18 studies [5, 7, 9–24] were included in the analysis. 8 studies [5, 7, 9–11, 13, 22, 24] were published in English, and the others were published in Chinese. 26 patients were involved in this review. 20 patients were from China, 2 were from India, and one each was from Japan, Turkey, Vietnam, and Egypt (Table 1).

**Table 1:** Clinical features, pathology of the kidney and lymph nodes and treatments administered of 26 patients with KD-associated nephrotic syndrome

Reported by	Case	Country	Gender	Age	Urine protein (g/24 h)	Serum albumin (g/L)	Serum creatinine(umol/L)	Serum IgE (IU/ml)	Eosinophils (%)	Renal biopsy	Treatment
X W Xiao	1	China	Male	23	12.77	34.6	88	582	0.44	Mesangial proliferation	Steroid+TW
X F Zhang	2	China	Male	46	6	26.3	62.9	43.4	0.15	Minimal change disease	Steroid
P C Li	3	China	Male	49	10.07	15.7	75		0.27	Mesangial proliferation	Steroid
H Sun	4	China	Male	40	8.34	30.8	480		0.15	Acute tubular necrosis	Steroid
Y W Zhang	5	China	Male	23	6.05	23		2500		IgA nephropathy	Steroid+CTX
L L Shao	6	China	Male	42	0.27		131	1316	0.354	IgA nephropathy	MMF
J L Meng	7	China	Male	43	6	15.5	115		0.574	Membranous nephropathy	Steroid
R Duan	8	China	Male	19		39.9	944	3000	0.154	FSGS	Steroid
	9	China	Femal	35	0.84	31.2		500	0.015	Mesangial proliferation	Steroid
C B Liu	10	China	Male	19	3.65		97.1	272	0.32	Membranous nephropathy	Steroid
	11	China	Male	21	5.32		185	397	0.17	IgA nephropathy	Steroid+TW
	12	China	Male	36	14.1		123.76	620	0.1	Membranous nephropathy	Steroid+CTX
	13	China	Male	25	12.8		88.4	989	0.44	Mesangial proliferation	Steroid+TW
	14	China	Male	14	8.65		53.04	172	0.056	Mesangial proliferation	Steroid+LEF
	15	China	Male	44	7.28		44.2	107	0.168	Mesangial proliferation	Steroid+LEF
	16	China	Male	53	6.99		176.8	160	0.122	Mesangial proliferation	Steroid+CTX
	17	China	Male	48	5.27		79.56	1378	0.26	Membranous nephropathy	Steroid
Y G Sha	18	China	Male	5	3.5	20.6	30	2000	0.21	Mesangial proliferation	Steroid
Koich	19	Japan	Male	9	11.1	12	238	4981	0.48	Minimal change disease	Steroid
Fatih	20	Turkey	Male	21	17.3	10	256	1987	0.21	FSGS	Steroid+CsA
Deepak	21	Vietnam	Male	14	4	21	123		0.11	Mesangial proliferation	Steroid
Chan	22	China	Male	25	19.7	18	85	1918		Mesangial proliferation	Steroid
S L Zhu	23	China	Male	23	9.3	16		1510	0.485	FSGS	Steroid
Mohamed	24	Egypt	Male	50	8.9	9	212		0.2	Membranous nephropathy	Steroid+MMF
K Sud	25	India	Femal	24	4.1	15	160			Minimal change disease	Steroid
Surendra	26	India	Male	18	8.6	21	62		0.03	Mesangial proliferation	Steroid

*TW* Tripterygium wilfordii, *CTX* Cyclophosphamide, *MMF* Mycophenolate mofetil, *LEF* Leflunomide, *CsA* Cyclosporine

Of the 26 patients included in the review, two were female and were from China and India, respectively, and the remaining patients were male. Thus, the male-to-female ratio was 12:1. Most of the patients presented with cervical lymphadenopathy or subcutaneous masses in the head or neck region. The mean age of these patients was 31.1 ± 14.2 years, the daily protein excretion level was 8.04 ± 4.62 g/24 h, the serum albumin concentration was 21.2 ± 8.57 g/L, the serum creatinine concentration was 170 ± 190 μmol/L, the blood eosinophil count was 23.7 ± 15.4%, and the IgE titer was 1285 ± 1223 IU/mL.

Renal biopsy was performed in all patients. Most of the patients presented with mesangial proliferative glomerulonephritis (13 patients). Other patients presented with membranous nephropathy (4 patients), minimal change disease (3 patients), focal segmental glomerulosclerosis (3 patients), IgA nephropathy (2 patients) and acute tubular injury (1 patient).

The majority of patients were treated with corticosteroids alone, while a small number of patients received corticosteroids combined with immunosuppressive agents, such as *Tripterygium wilfordii*, cyclophosphamide and leflunomide. With the exception of 2 patients who progressed to ESRD (end stage renal disease) and received hemodialysis, all other patients achieved remission with respect to their proteinuria. Simultaneously, the neck lymph nodes or masses became smaller. However, 5 patients experienced a proteinuria relapse during follow-up, including 4 patients with mesangial proliferative glomerulonephritis and one patient with membranous nephropathy.

## Discussion and conclusion

KD presents as a benign subcutaneous mass, and males are affected by KD more often than females. However, KD combined with kidney involvement is not rare. According to previous reports in the literature, kidney involvement often presents after the skin changes have been present for a few months. Regarding the patients who have kidney involvement associated with KD, 12–16% have proteinuria, and 59–78% have nephrotic syndrome [6, 25, 26]. The renal disease affecting the patients in our report was nephrotic syndrome.

Wang et al.[27]analyzed the clinical presentations of 20 patients with KD-associated nephrotic syndrome in China and found that men were predominantly affected by the disease, as the male: female ratio in their study was 19:1. The average proteinuria level in these patients was 8.67 ± 4.73 g/day. Renal biopsy was performed in 13 patients and showed that various forms of renal pathology were present. 9 patients had mesangial proliferative glomerulonephritis, 2 had MCD, and 2 had membranous nephropathy. Although mesangial proliferative glomerulonephritis was the most common disease demonstrated by renal biopsy in the review, MCD combined with acute renal tubular injury was demonstrated by renal biopsy in this report.

Although some patients with KD suffer from kidney disease, the pathogenesis of KD and its associated renal involvement is not very clear. Several studies of the immunopathogenic features of the disease found that the pathogenesis of KD with renal involvement may be related to high serum IgE levels and eosinophilia [9]. In addition, some authors have speculated that viral infections or toxins alter T cell immunoregulatory functions or induce IgE-mediated type I hypersensitivity, resulting in the release of lymphokines [11, 27]. As shown in the study by Liu C et al. [26], peripheral blood CD4+ and CD8+ T cell counts were significantly increased in patients with KD presenting with renal involvement, a finding that have may indicate Th cell immunoactivity. Th cells secrete cytokines to chemoattract B cells, mast cells and eosinophils and to promote the growth and differentiation of the cells at the site of an immune response.IL-4, which is expressed by T-helper 2 (Th2) cells and is not only an inducer of Th2 differentiation but also an eosinophil attractor, promotes B-cell class switching to IgE, resulting in high serum IgE levels. In addition, Katagiri et al. [28] found that TNF-α, IL-4, IL-5 and IL-13 mRNA levels in patients with KD were elevated before treatment but decreased markedly after surgery and radiation therapy. This observation supports the idea that Th2 cytokines play a part in KD development. According to Kimura et al. [29], T cells and mast cells play important roles in the pathogenesis of KD, a finding based on the observation that Th2-type cytokine and chemokine levels were increased in patients with the disease. These immunological triggers may result in the lymph node alterations characteristic of and the renal lesions associated with the disease.

Corticosteroids are particularly applicable for the treatment of KD with renal involvement [30], as they can improve proteinuria in patients with nephrotic syndrome by suppressing the transcription of inflammatory mediator genes [31]. However, termination of or reduction in steroid therapy often results in the recurrence of masses and proteinuria. Therefore, some patients require corticosteroids combined with immunosuppressive agents, such as cyclophosphamide and cyclosporine (CsA) [32–34]. Katagiri et al. [28] reported that CsA suppresses the activity of Th2 cytokines, which play an important role in KD development. Other therapies include irradiation and surgical removal. However, mass re-growth is common after surgical resection [35].

This report is about a singular manifestation of KD. Our patient, a middle-aged male, presented with atypical KD with renal involvement that responded well to steroids. The patient did not experience recurrence of either his skin lesions or his nephrotic syndrome during the follow-up period. We used corticosteroids alone, which successfully restored the patient’s renal function to normal. However, our follow-up time was short, and the long-term efficacy of the above treatment needs confirmation. In summary, steroids are still the first-choice treatment for KD with renal involvement but can be combined with immunosuppressive drugs if proteinuria persists.

MCD combined with acute renal tubular injury is rare in patients with KD presenting with renal involvement. Corticosteroids may be a beneficial treatment for renal injury in patients with KD.

## Abbreviations

ADA: Adenosine deaminase
CsA: Cyclosporine
ESRD: End-stage renal disease
KD: Kimura’s disease
MCD: Minimal change disease
